# Perceptions of plagiarism by biomedical researchers: an online survey in Europe and China

**DOI:** 10.1186/s12910-020-00473-7

**Published:** 2020-06-01

**Authors:** Nannan Yi, Benoit Nemery, Kris Dierickx

**Affiliations:** 1 Department of Medical Humanities, School of Humanities, Southeast University, Nanjing, 211189 China; 2 Centre for Biomedical Ethics and Law, Department of Public Health and Primary Care, KU Leuven, Leuven, Belgium; 3 Centre for Environment and Health, Department of Public Health and Primary Care, KU Leuven, Leuven, Belgium

**Keywords:** Plagiarism, Research misconduct, Biomedicine, University researchers, Europe, China

## Abstract

**Background:**

Plagiarism is considered as serious research misconduct, together with data fabrication and falsification. However, little is known about biomedical researchers’ views on plagiarism. Moreover, it has been argued – based on limited empirical evidence – that perceptions of plagiarism depend on cultural and other determinants. The authors explored, by means of an online survey among 46 reputable universities in Europe and China, how plagiarism is perceived by biomedical researchers in both regions.

**Methods:**

We collected work e-mail addresses of biomedical researchers identified through the websites of 13 reputable universities in Europe and 33 reputable universities in China and invited them to participate in an online anonymous survey. Our questionnaire was designed to assess respondents’ views about plagiarism by asking whether they considered specific practices as plagiarism. We analyzed if respondents in China and Europe responded differently, using logistic regression analysis with adjustments for demographic and other relevant factors.

**Results:**

The authors obtained valid responses from 204 researchers based in China (response rate 2.1%) and 826 researchers based in Europe (response rate 5.6%). Copying text from someone else’s publication without crediting the source, using idea(s) from someone else’s publication without crediting the source and republishing one’s own work in another language without crediting the source were considered as plagiarism by 98, 67 and 64%, respectively. About one-third of the respondents reported to have been unsure whether they had been plagiarizing.

Overall, the pattern of responses was similar among respondents based in Europe and China. Nevertheless, for some items significant differences did occur in disadvantage of Chinese respondents.

**Conclusions:**

Findings indicate that nearly all biomedical researchers understand (and disapprove of) the most obvious forms of plagiarism, but uncertainties and doubts were apparent for many aspects. And the minority of researchers who did not recognize some types of plagiarism as plagiarism was larger among China-based respondents than among Europe-based respondents. The authors conclude that biomedical researchers need clearer working definitions of plagiarism in order to deal with grey zones.

## Background

Integrity has been regarded as the foundation of scientific research [1, 2]. Unfortunately, research is sometimes conducted with violations of integrity [3–6]. Despite differences among various definitions of research misconduct, fabrication, falsification and plagiarism (FFP) are always included as severe deviations [7–9].

Unlike data fabrication and falsification, which are easily understood and universally viewed as reprehensible, plagiarism is more complex, both in theory and in practice, and it is, therefore, likely to be perceived variably by people, including scientists. The notion of plagiarism arose after the Renaissance, when in many fields individual intellectual work became more valued than before [10]. Henceforth, authors could be accused of plagiarism if they failed to give due credit to their predecessors. After hundreds of years, agreement emerged on how to define plagiarism: taking work (including words, images or ideas) from others without proper reference [11–13]. Nevertheless, although plagiarism seems to be thoroughly defined, experience suggests that grey zones of plagiarism remain.

It has been posited that people from different cultures have different understandings of what constitutes plagiarism [14–16]. Some scholars attributed the existence of divergences to differences in history, education and other factors between Western and other cultures [14, 16–18]. They argued that the notion of plagiarism originated from Western culture, since the time when words and ideas became valued as individual property, which might be perceived differently by other, mainly Asian, cultures [14, 19–21]. On the other hand, memorization and repetition have been very important in Chinese education and examinations, and students were encouraged to memorize and repeat literally the words of classic Confucian texts and other reputed historical persons, whereas proper citation (according to Western standards of referencing) was not emphasized [20, 22, 23]. With increasing intercultural communication, things have changed, but the influence of cultures cannot be ignored. A few empirical studies also revealed that Chinese scholars/students were not always capable of identifying certain forms of plagiarism [19, 21].

Some surveys and interview studies have investigated the influence of cultural factors on the perception of plagiarism. However, these studies mainly focused on students in universities [15, 16, 20, 24]. Since plagiarism is also a common problem among researchers and academics, it is important to test perceptions of plagiarism among scientific researchers of different cultures. Past studies among scientific researchers primarily examined the prevalence of plagiarism and attitudes towards specific forms of plagiarism [25, 26], as opposed to the very substance of plagiarism. As a consequence, little is known about the understanding of plagiarism among scientific researchers with different cultural backgrounds.

Considering the vast cultural difference between China and European countries, and the increasingly large number of scientific publications from China [3], the aim of our study was to investigate the understanding of plagiarism among biomedical researchers in Europe and China, and to explore if differences exist in the perception of plagiarism between both regions.

## Methods

### Survey instrument

#### Questionnaire design

We designed a questionnaire based on the TURNITIN definition of plagiarism [27], our review of documents on scientific integrity in Chinese universities [28] and an interview study of Chinese scholars based in Europe [29]. The survey contained three parts (see Additional file 1). Demographic information, such as age, gender, academic position, having a PhD degree and international research experience, was collected in the beginning of the questionnaire. The following section (Section 1) included questions regarding respondents’ general views about plagiarism, including factors determining their judgment whether an action constitutes plagiarism, and comparing the perceived seriousness of plagiarism with other forms of malpractice. The main section (Section 2) assessed the understanding of plagiarism, where several statements were divided into 7 categories based on their thematic similarity and respondents were asked to select the one(s) they thought constituted plagiarism.

#### Questionnaire elaboration

The questionnaire was elaborated using methodology similar to the study of Liao et al. [30]. A first version of the questionnaire was sent to three experts for their review and comments on structure and content. Based on their comments, the questionnaire was modified and then submitted to 14 researchers (philosophers, lawyers, medical doctors, ethicists, pharmacists, psychologists, biomedical scientists; originating from 6 different countries) at the Centre for Biomedical Ethics and Law, KU Leuven, for further refinement.

The original survey was designed in English. Since the survey also targeted Chinese researchers, the questionnaire was translated into Chinese by the first author NY. To validate the language, the translated questionnaire and the original questionnaire were sent for review to three Chinese doctoral and postdoctoral biomedical researchers, who had worked in international research environments. Additional minor changes were made according to their comments.

Based on the suggestions received from the respondents after the first release of the online survey, we further improved the questionnaire and added the option of “none of the above” for each question in Section 2.

### Selection and invitation of respondents

Our study was targeted at biomedical researchers (i.e. researchers active in medicine, pharmaceutical sciences and life sciences) based at leading research universities in Europe and China. We selected 13 universities from *the League of European Research Universities* (based on whether they had medical schools and regional spread) [31, 32] and 33 universities with biomedical schools from *Class A Universities of the Double First Class University in China* (see Additional file 2) [33, 34]. The first author (NY) manually retrieved the e-mail addresses of all researchers (professors, associate professors, assistant professors, postdoctoral researchers) whose email addresses were available on the university websites. (Other people, such as doctoral or master students, and administrative staff were also included if their academic position was not clearly indicated on the website, but their academic positions were inquired about by our questionnaire.)

Using her personal KU Leuven e-mail address, the first author NY sent invitation e-mails (with “Invitation to the survey of plagiarism definition” as the subject of the e-mail) (see Additional file 3) explaining the purpose of the study and providing a link to the online questionnaire to all the target researchers (except for the researchers based at KU Leuven, where the invitation was sent by the university). Europe-based researchers received the invitation in English and China-based researchers received the invitation in both English and Chinese. E-mails were sent in bulks grouped by university but without the names of recipients being visible. The anonymity of participation was guaranteed. Reminders were sent after 2 and 4 weeks (except for KU Leuven, where only one reminder was sent by the university after 2 weeks). All data were collected from March 2018 to July 2018.

### Ethics, consent and permissions

The study was approved by the Social and Societal Ethics Committee of the KU Leuven (dossier G- 2017 08885).

Filling out the survey counts as informed consent to participate in this study, which was clearly indicated in the online questionnaire and approved by the Social and Societal Ethics Committee of the KU Leuven.

### Statistical methodology

Summary data are presented as means and standard deviations for continuous variables and as percentages (the number of respondents selecting each option/the total number of valid responses× 100) for categorical variables.

In general, the null hypotheses were that the proportions of responses to the questions would not differ between the groups of European and Chinese respondents.

Comparisons of percentages between the European and Chinese universities were performed using the Chi square test for binary variables and categorical variables (gender, mother tongue, current academic position, PhD degree, year of obtaining PhD degree (in 10-year categories) and international research experience) or Mann-Whitney U test for continuous variables (age). Logistic regression models and proportional odds models were used to compare Europe-based and China-based respondents for binary variables and ordinal variables, correcting for demographic differences. These models were also used to analyze the association of demographic factors with responses to the other questions. Values of odds ratios (ORs) and their 95% confidence intervals (CIs) were calculated. The null hypothesis was rejected when a two-tailed *P* value was less than 0.05. For three demographic factors: gender, current academic position and year of PhD, the two other factors were corrected when analyzing the association of one factor with the responses to questions in Section 1 and 2.

For questions of Section 2 in each group (see Additional file 1), the kappa coefficient was determined as a measure of consistency within each respondent between the responses to different questions.

Data Analyses were performed using SAS 9.4.

Reporting of this study follows the STROBE statement [35].

## Results

For the first round of surveys, we sent e-mails to 25,648 biomedical researchers based at leading universities in China and Europe, and 1397 emails were bounced back. These addresses to which emails were not delivered successfully were regarded as invalid addresses. For the second and the third round, we sent reminder emails to the other 24,251 valid addresses and eventually collected valid responses from 1030 respondents (total response rate 4.2%), 826/14,757 (5.6%) from Europe and 204/9494 (2.1%) from China. The numbers of respondents who answered some questions about demographic characteristics do not add up to 1030 because some invalid answers were excluded. Only complete responses and responses with fewer than two invalid answers were included, and analyzed as valid responses. The exact numbers are shown in Table 1 and Table 2.

**Table 1:** Demographic characteristics of the respondents

Variables	Percentage of total respondents (%)	Percentage of researchers in Europe (%)	Percentage of researchers in China (%)	*P* value^a^
**Age (*n*** **= 1026)**				
< =30y	10.3	11.0	7.4	<.001
31-40y	33.7	32.3	39.6	
41-50y	24.7	22.3	34.1	
51-60y	20.6	21.6	16.3	
> 60y	10.7	12.7	2.5	
**Gender (*n*** **= 1029)**				
Female	41.6	44.6	29.4	<.001
Male	58.4	55.4	70.6	
**Mother tongue (*n*** **= 1030)**				
Chinese	21.3	2.7	96.6	<.001
English	13.3	16.1	2.0	
Other	65.4	81.2	1.5	
**Current academic position (*n*** **= 1030)**				
Professor	29.7	25.2	48.0	<.001
Associate professor	21.6	19.2	30.9	
Assistant professor	9.8	10.6	6.4	
Postdoc	20.4	24.5	3.9	
Other	16.3	18.5	7.4	
Not a scientific researcher	2.2	1.9	3.4	
**PhD degree (*n*** **= 1030)**				
Yes	84.3	82.6	91.2	0.001
Current PhD candidate	7.4	8.8	1.5	
No	8.4	8.6	7.4	
**Year of obtaining PhD degree (*n*** **= 828)**				
< 1979	2.3	2.9	0.0	<.001
1979–1988	6.4	7.5	2.3	
1989–1998	19.4	22.0	9.8	
1999–2008	33.0	28.3	50.6	
2009–2018	38.9	39.3	37.4	
**International research experience (> 6 months) (*n*** **= 1030)**				
Yes	62.4	62.1	63.7	0.669
No	37.6	37.9	36.3	

^a^*P* values based on Chi square tests when comparing Europe and China

**Table 2:** Percentage of respondents who regarded the practice as plagiarism

Statement of practice	Percentage of total respondents (%, *n* = 1030)	Percentage of researchers in Europe (%, *n* = 826)	Percentage of researchers in China (%, *n* = 204)	*P* value^a^	Adjusted OR (95% CI)^b^
**Statement 17. Appropriation of others’ text, image and ideas**					
a. Copying text from someone else’s publication without crediting the source.	97.7	98.7	93.6	<.001	**0.15 (0.05;0.43)**
b. Copying text from someone else’s publication with crediting the source, but without quotation marks.	48.5	51.6	36.3	<.001	**0.40 (0.27;0.59)**
c. Copying text from someone else’s publication with crediting the source and with quotation marks.	6.1	6.2	5.9	0.876	1.09 (0.51;2.36)
d. Copying an image from someone else’s publication without crediting the source.	96.0	96.4	94.6	0.249	0.54 (0.22;1.34)
e. Using idea(s) from someone else’s publication without crediting the source.	67.1	67.4	65.7	0.634	1.03 (0.70;1.53)
**Statement 18. Appropriation of online sources**					
a. Copying text from an online source without crediting the source.	95.5	97.3	88.2	<.001	**0.20 (0.09;0.45)**
b. Copying text from an online source that has no list of authors, and without crediting the source.	79.2	81.7	69.1	<.001	**0.46 (0.30;0.71)**
**Statement 19. Rephrasing or summarizing another person’s work**					
a. Rephrasing another person’s work without crediting the source.	84.6	83.8	87.8	0.160	1.27 (0.75;2.17)
b. Rephrasing text from someone else’s publication without significant modification of the original, but with crediting the source.	16.9	17.7	13.7	0.178	0.77 (0.46;1.29)
c. Summarizing another person’s work without crediting the source.	78.6	80.5	71.1	0.003	0.68 (0.44;1.04)
**Statement 20. Text resources of article writing**					
a. Paying someone else to write a paper without granting authorship.	37.3	33.5	52.4	<.001	**2.44 (1.67;3.55)**
b. Having someone else to write a paper for free without granting authorship.	49.5	46.0	63.7	<.001	**2.18 (1.50;3.17)**
c. Putting together pieces from different publications, and presenting the result as one’s own work.	94.4	95.0	91.7	0.062	0.50 (0.24;1.04)
d. When writing a literature review, using the same framework of others’ review, without crediting the source.	53.1	53.0	53.4	0.917	1.01 (0.70;1.45)
e. With permission from the original author, using another’s text without crediting the source.	67.4	68.9	61.3	0.038	**0.65 (0.44;0.96)**
**Statement 21. Publishing in multiple languages**					
a. Republishing others’ work in another language without crediting the source.	98.4	98.4	98.5	0.915	1.47 (0.34;6.38)
b. Republishing one’s own work in another language without crediting the source.	64.2	67.7	50.00	<.001	**0.38 (0.26;0.56)**
**Statement 22. Reuse of research proposal**					
a. Reusing one’s own previously rejected research proposal for another funding application without crediting the source.	11.2	9.3	18.6	<.001	**2.17 (1.27;3.70)**
b. Reusing a significant portion of one’s own previous publication for a new publication without crediting the source.	79.0	79.4	77.4	0.536	0.65 (0.41;1.01)
**Statement 23. Republication of dissertations**					
a. One has submitted work as dissertation/thesis, and submits parts of it to a journal afterwards without crediting the source.	29.2	32.4	16.2	<.001	**0.44 (0.28;0.70)**
b. One has submitted work as dissertation/thesis, and submits a summary of it to a journal afterwards without crediting the source.	26.3	29.5	13.2	<.001	**0.43 (0.26;0.70)**

* *P* values based on Chi square tests when comparing Europe and China

** ORs (with 95% CIs) based on logistic regression analysis, with adjustments for demographic variables (including age, gender, academic position, PhD, international research experience). Reference is Europe

### Demographic information

Table 1 displays demographic and academic characteristics of the respondents. The values of mean and standard deviation for age (in years) of respondents in Europe and China were 45.2 ± 12.3 and 42.6 ± 8.6, respectively. The majority of respondents were aged 31–60 years, with the proportion of this age category being larger in China. There were more male respondents (58.4%), with a higher proportion in China (70.6%). A minority of respondents had English as their mother tongue (16% in Europe and 2% in China).

The respondents were mainly professors (29.7%) and associate professors (21.6%). More than 80% of the respondents had a PhD degree, typically obtained since 1999 (71.9%). More than 60% of the respondents in both regions had international research experience of more than 6 months, with no difference between Europe and China.

In view of the demographic differences between respondents from Europe and China, we conducted all logistic regression analyses with adjustments for those variables (including age, gender, academic position, PhD and international research experience) to yield adjusted odds ratios (aORs) with 95% confidence intervals (CIs), always taking Europe-based respondents as the reference category.

### Perceptions of plagiarism practice

The majority of respondents from both regions successfully identified most statements of plagiarism (Table 2). Nevertheless, in several instances the likelihood of not perceiving some practices as plagiarism did differ significantly between respondents in Europe and China. Most odds ratios were not materially affected by adjusting for demographic variables, except for one statement (**summarizing another person’s work without crediting the source**) for which significance between European and Chinese respondents disappeared after adjustment.

Almost all respondents in both Europe (98.7%) and China (93.6%) considered **copying text without crediting the source** as plagiarism. Nevertheless, the minority of respondents who did not successfully recognize this most obvious form of plagiarism was considerably larger (five-fold) for respondents from China (6.4%) than for respondents from Europe (1.3%), thus yielding a highly significant aOR of 0.15 (95%CI 0.05;0.43) (Europe being taken as the reference). For copying **images**, the difference between China (5.4%) and Europe (4.6%) did not reach significance (aOR 0.54, 95% CI 0.22;1.34). **Using someone else’s ideas without giving credit** was considered as plagiarism by two thirds (67%) of respondents, again without difference between China and Europe (aOR 1.03, 95% CI 0.70:1.53)**. Copying text with crediting the source, without quotation marks** was regarded as plagiarism by half of the total respondents, with China-based respondents being less likely to do so (aOR 0.40, 95%CI 0.27;0.59).

Compared to copying text, respondents were generally more lenient about improper rephrasing, especially close rephrasing. **Rephrasing another person’s work without crediting the source** and **summarizing another person’s work without crediting the source** were perceived as plagiarism by around four fifths of the respondents, without differences between China-based and Europe-based respondents (after adjustment for demographic characteristics). In contrast, almost one fifth of the respondents indicated **rephrasing text from someone else’s publication without significant modification of the original, but with crediting the source** as plagiarism**.**

With respect to online sources, the majority (96%) of the respondents reported **copying from an online source without crediting the source** as plagiarism, while fewer (79%) reported **copying from an online source with no list of authors, and without crediting the source** as plagiarism. In contrast to respondents in Europe (97.3 and 81.7%, respectively), respondents in China (88.2 and 69.1%, respectively) were significantly less likely to consider these practices as plagiarism, yielding aORs of 0.20 and 0.46, respectively.

Perceptions about resources of article writing were examined as well. A substantial number of respondents did report that having someone else write a paper constituted plagiarism, but it depended on the payment: almost half of the respondents indicated that **having someone else to write a paper for free without granting authorship** was plagiarism, while fewer (37%) indicated that **paying someone else to write a paper without granting authorship** constituted plagiarism. In addition to these two practices, nearly one third of the respondents did not consider **using another’s text without crediting the source, but with permission from the original author** as plagiarism. Compared to respondents in Europe (34 and 46%), more respondents in China (52 and 64%) tended to indicate that **paying someone else to write a paper without granting authorship** and **having someone else to write a paper for free without granting authorship** constituted plagiarism, with significant aORs of 2.44 and 2.18.

Publishing in multiple languages was also perceived differently. Almost all respondents indicated **republishing others’ work in another language without crediting the source** as plagiarism (98%), while less than two thirds considered **republishing one’s own work in another language without crediting the source** as plagiarism. In contrast to respondents in Europe (68%), fewer respondents in China (50%) tended to identify **republishing one’s own work in another language without crediting the source** as plagiarism, yielding a significant aOR of 0.38.

### General views about plagiarism

In addition to the specific practices above, we also explored some other aspects of plagiarism.

**Fig. 1:**
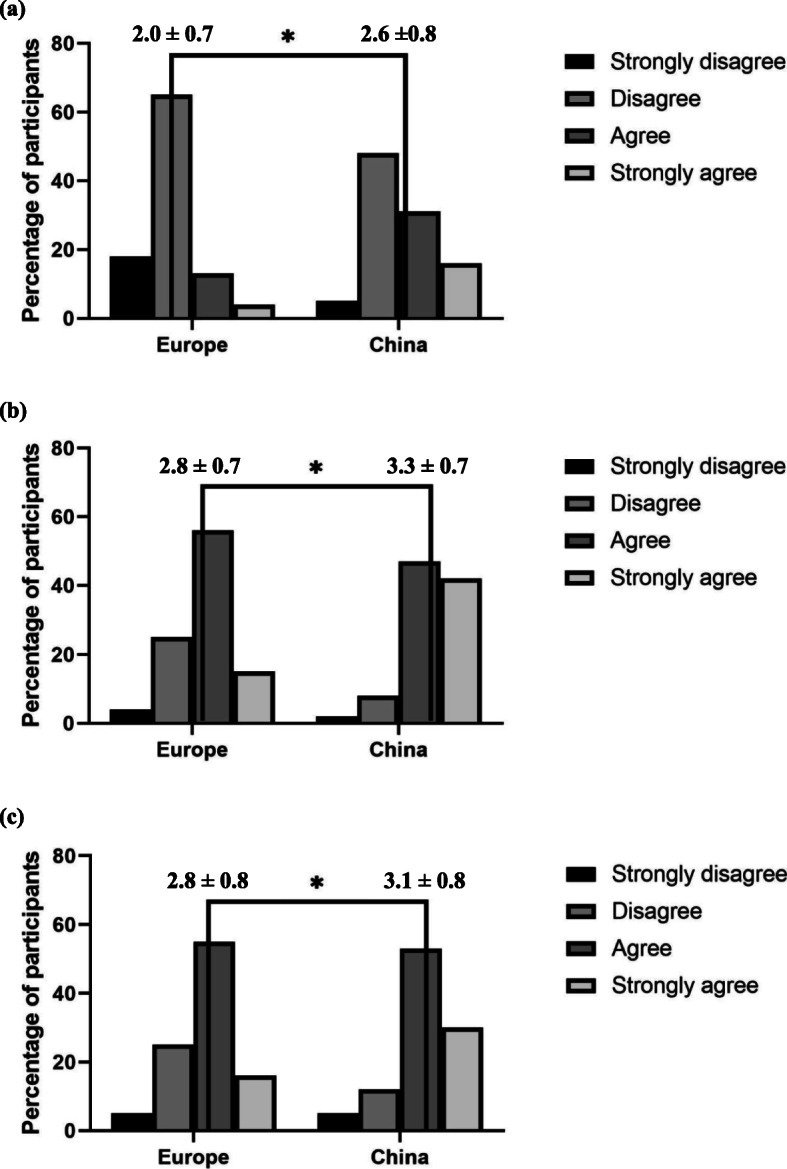
**a** Attitudes towards the statement “**Plagiarism is a greater threat to biomedical research than data falsification**”. * *P* < 0.001 (Mann-Whitney test); Strongly disagree = 1, Disagree = 2, Agree = 3, Strongly agree = 4; Means and standard deviations (SD) of the scores are presented as: mean ± SD. **b** Attitudes towards the statement “**Plagiarism is a greater threat to biomedical research than granting co-authorship to someone whose contribution doesn’t justify it**”. * *P* < 0.001 (Mann-Whitney test); Strongly disagree = 1, Disagree = 2, Agree = 3, Strongly agree = 4; Means and standard deviations (SD) of the scores were presented as: mean ± SD. **c** Attitudes towards the statement “**Plagiarism is a greater threat to biomedical research than submitting a manuscript to more than one journals simultaneously**”. * *P* < 0.001; Strongly disagree = 1, Disagree = 2, Agree = 3, Strongly agree = 4; Means and standard deviations (SD of the scores were presented as: mean ± SD

In general, the respondents indicated that plagiarism was a bigger threat to biomedical research than **granting co-authorship to someone whose contribution doesn’t justify it** and **submitting a manuscript to more than one journal simultaneously**, but a lesser threat than **data falsification** (Fig. 1 a-c). Compared to their counterparts in Europe, the respondents in China showed a stricter attitude.

Respondents’ other views about plagiarism are presented in Table 3. Perceived factors that **determine whether a body of copied and unattributed text constitutes plagiarism** were inquired in the survey. About 6% of the respondents reported that the practice of plagiarism did not depend on the **intention**, the **length** or the **part of the copied text**. In contrast, three-quarter of the respondents (75%) indicated that **intention** should be considered when deciding whether a practice is plagiarism or not. Half of the respondents indicated that the **length** (53%) and the **part** (45%) of the copied text did matter. In contrast to respondents in Europe, those in China tended to agree more frequently (55% vs 43%, aOR 1.91) that **part of the copied text** determined whether a practice was plagiarism or not (*p* < 0.05). On the contrary, compared to respondents in Europe (77%), those in China (67%) tended to agree less frequently that the **intention** mattered (aOR 0.59).

**Table 3:** Percentage of respondents who selected each option

Question	Percentage of total respondents (%, *n* = 1030)	Percentage of researchers in Europe (%, *n* = 826)	Percentage of researchers in China (%, *n* = 204)	*P* value^*^	Adjusted OR (95% CI)^**^
**Question 15. Which factor(s) do you think decides whether a body of copied and unattributed text constitutes plagiarism or not?**					
a. The length of the copied text	53.0	51.3	59.8	0.030	1.24 (0.86, 1.80)
b. The part of the copied text	45.2	42.6	55.4	0.001	1.91 (1.32, 2.77)
c. The presence of an intention to copy without attribution	75.3	77.4	67.2	0.002	0.59 (0.39, 0.89)
**Question 16. Have you ever been unsure whether you are plagiarizing?**					
a. Yes	31.3	34.5	18.1	<.001	0.41 (0.26, 0.64)

^a^*P* values based on Chi square tests when comparing Europe and China

^b^ ORs (with 95% CIs) based on logistic regression analysis, with adjustment for demographic variables (including age, gender, academic position, PhD, international research experience). Reference is Europe

At the practical level, nearly one third of the respondents stated to have been unsure whether they had been plagiarizing. Compared with respondents in Europe (34%), respondents in China (18%) were significantly less likely to doubt whether they had been plagiarizing (aOR 0.41).

### Association with demographic factors

The responses to some questions were associated with some demographic factors, such as age, gender, mother tongue and (year of) PhD degree (Table 4), which justified the necessity of correction for these factors when comparing responses in China and Europe (as presented in Table 2 and Table 3).

**Table 4:** Associations with demographic characteristics of all respondents (OR values were presented)

Question/Statement	Age (a)	Gender (b)	Mother tongue (c)	Current academic position (d)	PhD degree (e)	Year of PhD degree (f)	International research experience (g)
**Question 12. Greater threat than data falsification**	1.01*	1.44*	4.70(CH) 0.52(E)	√	1.73(N)		1.03
**Question 13. Greater threat than granting co-authorship**	0.99*	0.77	4.65(CH)	√		√	1.08
**Question 14. Greater threat than submitting to more than one journal**	0.98*	0.96	2.66(CH)			√	0.97
**Question 15. Factors deciding plagiarism**							
a. length of the copied text	1.01	0.81	1.42(CH)		0.50(CP)		1.03
b. part of the copied text	1.01	0.87	1.68(CH) 0.56(E)				1.07
c. intention to copy without attribution	1.00	0.78	0.64CH)				0.88
**Question 16. Have been unsure whether oneself is plagiarizing**	0.98*	0.91	0.43(CH)		2.24(CP) 1.88(N)		1.02
**Statement 17. Appropriation of others’ text, image and ideas**							
a. Copying text from someone else’s publication without crediting the source.	1.00	1.77	0.20(CH)				0.59
b. Copying text from someone else’s publication with crediting the source, but without quotation marks.	0.98*	1.03	0.62(CH)			√	0.88
c. Copying text from someone else’s publication with crediting the source and with quotation marks.	1.00	1.03					0.70
d. Copying an image from someone else’s publication without crediting the source.	0.98	2.05			0.32(N)		0.56
e. Using idea(s) from someone else’s publication without crediting the source.	1.00	1.44*					1.04
**Statement 18. Appropriation of online sources**							
a. Copying text from an online source without crediting the source.	0.99	2.03	0.24(CH)				1.26
b. Copying text from an online source that has no list of authors, and without crediting the source.	1.00	0.83	0.58(CH) 1.86(E)				1.09
**Statement 19. Rephrasing or summarizing another person’s work**							
a. Rephrasing another person’s work without crediting the source.	1.00	1.41					0.96
b. Rephrasing text from someone else’s publication without significant modification of the original, but with crediting the source.	1.00	1.37					0.99
c. Summarizing another person’s work without crediting the source.	1.02*	1.55*	0.52(CH)				1.31
**Statement 20. Text resources of article writing**							
a. Paying someone else to write a paper without granting authorship.	0.99	0.90	2.42(CH)	√	1.79(CP)		1.17
b. Having someone else to write a paper for free without granting authorship.	0.99	1.01	2.19(CH)	√			1.08
c. Putting together pieces from different publications, and presenting the result as one’s own work.	1.03*	1.83					0.63
d. When writing a literature review, using the same framework of others’ review, without crediting the source.	1.02*	1.24	0.60(E)		0.56(CP)		0.87
e. With permission from the original author, using another’s text without crediting the source.	1.01	1.25	0.70(CH)				0.93
**Statement 21. Publishing in multiple languages**							
a. Republishing others’ work in another language without crediting the source.	0.99	1.98					0.60
b. Republishing one’s own work in another language without crediting the source.	1.01	0.99	0.51(CH)		0.56(N)		0.89
**Statement 22. Reuse of research proposal**							
a. Reusing one’s own previously rejected research proposal for another funding application without crediting the source.	0.99	0.94	2.21(CH) 0.34(E)		2.08(N)	√	1.08
b. Reusing a significant portion of one’s own previous publication for a new publication without crediting the source.	1.00	1.54*			0.60(N)		0.82
**Statement 23. Republication of dissertations**							
a. One has submitted work as dissertation/thesis, and submits parts of it to a journal afterwards without crediting the source.	1.00	1.07	0.50(CH) 0.58(E)		1.71(N)		1.22
b. One has submitted work as dissertation/thesis, and submits a summary of it to a journal afterwards without crediting the source.	1.01	1.20	0.38(CH) 0.62(E)		1.92(N)		1.35*

(a)(b) * There is association between the response and the demographic factor

(b) “Male” as the reference

(c) “Other” as the reference. “CH” stands for Chinese, and “E” stands for English. Only odds ratios with statistical significance are listed

(d) (f) “√” indicates association between responses to the statement/question and the demographic factor

(e) “PhD” as the reference. “CP” stands for “currently a PhD candidate”, and “N” stands for having no PhD degree. Only odds ratios with statistical significance are listed

(g) “With international research experience of more than 6 months” as the reference

More specifically, responses to many questions differed with the mother tongue. For example, compared with the other language (neither English-native nor Chinese-native) speakers, Chinese-native speakers were more likely to agree that **plagiarism was a greater threat to biomedical research** than the other given malpractices – data falsification, gift authorship and multiple submission – but they were less sensitive to plagiarism of **online sources**. They were also less likely to perceive that **the intention decided whether or not a body of copied and unattributed text constitutes plagiarism**.

Moreover, other factors also had effects on the responses. As age increased, participants were less likely to doubt **whether they themselves had committed plagiarism**. Researchers without a PhD degree were more likely to doubt **whether they themselves had been plagiarizing.** Researchers without a PhD degree (not a PhD candidate, either) had more concerns with republication of dissertations and misuse of one’s previously research proposals and less sensitivity towards **copying an image from someone else’s publication without crediting the source** and **reusing a significant portion of one’s own previous publication for a new publication without crediting the source.**

Current academic position and year of obtaining PhD proved to associate with understanding of a few practices as well.

### Agreement analysis

Agreement analysis was performed for questions in the same group in Section 2 to explore the consistency within each respondent between perceptions of different practices. Despite the little internal consistency among the responses to most practices, we did observe agreement between a few practices, as follows (More data is available in Additional file 4). Respondents who considered **paying someone else to write a paper without granting authorship** as plagiarism, also tended to consider **having someone else to write a paper for free without granting authorship** as plagiarism (Kappa coefficient = 0.71).

Regarding republication of a dissertation/thesis, respondents who indicated **one has submitted work as dissertation/thesis, and submits parts of it to a journal afterwards without crediting the source** as plagiarism, also tended to indicate **one has submitted work as dissertation/thesis, and submits a summary of it to a journal afterwards without crediting the source** as plagiarism (Kappa coefficient = 0.78).

## Discussion

Plagiarism has been consistently regarded as a severe type of research misconduct, on a par with fabrication and falsification [10–12]. Many scholars have discussed the nature and harm of plagiarism, such as violation of integrity, intellectual property, infringement of the copyright, monetary loss of others [36–38]. Definitions, reasons, prevalence of and attitudes towards plagiarism have also been studied [21, 39–43]. However, when we discuss and address plagiarism worldwide, one question appears to have been somehow ignored: How do scientists understand plagiarism? The present study aimed to provide some insights into this question. Through an online survey, we investigated if biomedical researchers in Europe and China differed in their perceptions of certain practices as plagiarism. Our findings revealed a range of perceptions of plagiarism in both Europe and China. In terms of prevalences of answers to most of the questions on plagiarism, the differences between China-based and Europe-based respondents were not impressive, indicating that the knowledge and perception about plagiarism were largely similar in the two groups. Nevertheless, when looking at adjusted ORs, the likelihood of perceiving things differently proved substantial for some practices. In other words, the minority of respondents with a “deviant” response proved substantially larger among China-based respondents compared to Europe-based respondents in some instances. The findings also suggest that despite a good understanding of plagiarism by most respondents, knowledge of plagiarism is still lacking among some respondents, especially regarding certain subtle forms of plagiarism.

### Differences between respondents in Europe and China

Respondents in China were significantly less likely than respondents in Europe to consider the following five practices as plagiarism, even though four of these practices are regarded as plagiarism by internationally agreed guidance or policy [11, 12]: copying unattributed text from someone else’s publication (aOR 0.15); copying attributed text from someone else’s publication, but without quotation marks (aOR 0.40); copying text from an online source without crediting the source (aOR 0.20); copying text from an online source that has no list of authors, and without crediting the source (aOR 0.46) and submitting parts of or a summary of one’s previous thesis (aOR 0.44 and 0.43, respectively). To our knowledge, republishing from one’s thesis is not always treated as plagiarism, but the differences between respondents in both regions might reflect the divergently rigorous and prudent attitudes in scientific publishing.

Republishing one’s own work in another language without referencing was also less likely to be perceived as plagiarism by respondents in China (aOR 0.38). Of note, one third of Europe-based researchers in our survey also did not view this practice as plagiarism. Yet, unacknowledged duplicate publication in another language is deemed as self-plagiarism or duplicate (dual) publication [8, 44, 45], which is unequivocally an undesirable practice. Some Chinese scholars republish their work in English after successfully publishing the same work in Chinese, so as to gain more publications and increase their competitiveness in promotion [46]. In their empirical analysis, Tucker et al. [47] also observed substantial overlap with Chinese published work in around one fifth of the English manuscripts from Chinese institutions. In this regard, it is not surprising, according to our findings, that only half of the respondents in China considered duplicate republication in another language as plagiarism. This agrees with the survey of Pupovac et al., where about half of the students surveyed found that self-plagiarism was “harmless” or “justified” [43].

On the contrary, paid or unpaid ghostwriting was regarded as plagiarism by *more* respondents in China than in Europe (aOR 2.44 and 2.18, respectively). As noted by previous reports, ghostwriting and online ghostwriting transactions are not rare in China [46, 48], especially among medical researchers, including doctors, whose promotion relies on publication of SCI papers rather than on the number of patients they see [46]. We speculate that Chinese scholars in our sample were more likely than European scholars to consider ghostwriting as objectionable, because they were more exposed not only to ghostwriting and ghostwriting transactions, but also to negative reports about the practice.

Surprisingly, on the one hand, respondents in China were more likely than respondents in Europe to agree that plagiarism was a greater threat to biomedical research than the other three misbehaviors listed in the questionnaire and they also appeared more self-confident in terms of the understanding of plagiarism. However, on the other hand, they were less likely to detect a few specific practices as plagiarism. This indicates that, in general, respondents in China did realize that plagiarism was unacceptable, but the awareness of what constitutes plagiarism was poor in a larger proportion of respondents. Our findings differ from a recent study by Li et al. [49], which observed a higher acceptance of plagiarism among Chinese scientific researchers. The discrepancy with the latter study may be due to the different academic domains and different institutions of the respondents in these two studies.

Nevertheless, we cannot conclude (nor exclude) from our survey that “cultural differences” between Chinese and western societies play a role in how plagiarism is perceived by Europe-based and China-based researchers. To further investigate the cultural influence, a more profound “anthropological” approach would be needed in future studies.

In addition to the differences above, the similarities of “correct/incorrect” responses to other questions in both regions are also worth noting.

### Perceptions of specific practices

#### “Copying and pasting text” and related practices

As expected, “copying and pasting text without crediting the source” was perceived as plagiarism by the vast majority of respondents. Nevertheless, in the views of some respondents, certain conditions protected the practice of copying from being plagiarism: copying text from someone else’s publication with crediting the source, but without quotation marks; using another’s text without crediting the source, but with permission from the original author and rephrasing text from someone else’s publication without significant modification of the original, but with crediting the source. Without proper referencing, readers would regard the copied material as original text of the new author(s), which should be deemed as plagiarism [50]. Moreover, paraphrasing without giving credit has been considered undesired by many scholars [51–53]. Compared with simple “copying and pasting”, it is more difficult to detect this type of plagiarism with text-matching software and other strategies have to be used. Hence, proper citations, and quotations in some cases, are needed to indicate the origin of the text.

In addition, although most of the surveyed scientists considered copying text without crediting the source as plagiarism, the perception changed with regard to citing from online sources. Fewer respondents considered improper referencing of online sources in the absence of identifiable authors as plagiarism. Other studies have made similar observations. In a survey in 2011, not all university instructors surveyed identified copying texts or images from online sources and close paraphrasing as “definitely plagiarism” [54]. When the definition of plagiarism is not clear to instructors, students in universities are very unlikely to gain correct understanding of plagiarism.

#### Other practices

Compared to the practices above, our study discovered no better understanding of other relevant behaviors, such as plagiarism of ideas.

Appropriation of idea(s) without crediting the source, which is clearly defined as plagiarism [8, 12, 13], was not identified as plagiarism by a substantial minority (33%) of the respondents. This finding demonstrates that not all respondents had correct and adequate knowledge of basic definitions of plagiarism. Some previous studies stated that the value and protection of ideas differed across cultures and that protection of ideas was lacking in Asian countries, including China, where harmony and conformity were valued more than uniqueness [55]. However, our results did not observe this difference between Western and Asian cultures with regard to plagiarism of ideas. More in-depth investigation of this, admittedly, not straightforward issue of plagiarism of ideas might show different results.

### Other views

In general, most respondents considered plagiarism to be more threatening to biomedical research than gift authorship and multiple submission, and less threatening than data falsification. This finding is consistent with the study of Roberts et al. [56], where plagiarism was ranked between data fabrication and inappropriate authorship in terms of severity. One possible reason might be that plagiarism does not affect the authenticity of data [2]. In a study of Bouter et al., attendees of international research integrity conferences were invited to rank 60 research misbehaviors [57]. In spite of a perceived higher prevalence, plagiarism ranked behind data fabrication and falsification in terms of its impact on truth [57].

Concerning factors that determine plagiarism, most respondents indicated that intention mattered. Though there might be different motivations behind unintentional and intentional plagiarism, it is always difficult to detect intention, so this factor is not often considered relevant by guidance or policies about plagiarism [8, 9, 58]. As a consequence, better strategies to address unintentional plagiarism should be designed.

Since the meaning of plagiarism is not always clearly understood, it is possible that some people did not know whether their own behaviors constituted plagiarism or not, which was supported by our findings: nearly one third of the respondents reported to have had moments doubting whether they had been plagiarizing. This shows that plagiarism is not always “black and white”, and that grey zones persist (in contrast perhaps to fabrication and falsification, where things are more clearcut). This should be taken into account by those in charge of teaching or judging scientific integrity. Surveys in other regions also revealed the existence of confusion about what constitutes plagiarism [18, 59, 60]. Hence, systematic education on scientific writing and proper citations is needed [59, 61].

### Influences of demographic factors

We found associations between mother tongue and responses to many questions. For example, compared with the group of other language speakers (whose mother tongue is neither English nor Chinese), more Chinese-native speakers tended to agree that length and part of the copied text mattered, while English-native speakers were less likely to think part mattered. This difference might be due to different levels of difficulty experienced when writing scientific articles in English, which is also supported by Biagioli [62]. As a consequence, the different proportions of English-native speakers in our respondents in Europe and China might have contributed to the different responses observed between both regions. Thus, we found that Chinese-native speakers tended to believe more frequently than English-native speakers that the type of copied text mattered, which was in accordance with the viewpoint held by some non-native English speakers in Biagioli’s study: appropriation of text in certain parts (such as the Introduction Section) from Anglophone papers would help, as long as the result is original [62]. Our study and that of Biagioli indicate that non-native English speakers understand the notion plagiarism differently from native speakers.

Our female respondents showed slightly more concerns with several practices, including idea plagiarism and self-plagiarism. Although several studies did not observe gender differences regarding perceptions or prevalence of research misconduct [61, 63], some scholars detected a difference [64–67] and attributed it to social and psychological differences between males and females [68, 69].

Having a PhD degree was associated with some responses. Liao et al. [30] found that participants with a PhD degree did not differ from other participants on their opinions towards academic misconduct. Nonetheless, in our present study, researchers with a PhD degree proved to be more self-confident with their understanding of plagiarism. In contrast, researchers without a PhD or not currently pursuing a PhD degree had a poorer understanding of image plagiarism and self-plagiarism, and more concerns with republishing one’s thesis and reusing one’s previously rejected research proposals. The differences above might be partly explained by the experience with practices and training on research when performing one’s PhD research project [70].

Moreover, confidence increased with age. With more working years and research experience, senior researchers might have a better understanding of plagiarism. It has been reported that as years of study and working increased, tolerance toward plagiarism increased [69] while incidence of research misconduct declined [59, 65]. Nevertheless, another study also pointed out that the knowledge about plagiarism did not increase with age and years of study [67]. As a consequence, we cannot overlook the importance of research integrity training for senior researchers.

In light of the fact that tolerance toward plagiarism varied across countries [71], it is surprising that international research experience of more than 6 months was hardly associated with understanding of plagiarism. One possible reason might be that the length of stay is not long enough to have any effect on researchers’ understanding of plagiarism. In addition, the questionnaire did not investigate the countries in which the international research was conducted. It is possible that positive and negative effects were mixed, therefore no effect was observed in the present study.

### Limitations

Compared to some other online surveys that also investigated plagiarism or other aspects of research integrity [61, 72–74], we had a low response rate (4.2%). Despite the low numbers of respondents, we did observe a number of significant differences for some questions. The large geographical span might be one contributing factor of our low response rate. Besides, considering the large number of our target respondents, we sent invitation emails in bulks, which might have directed the invitation to the spam box of some potential respondents. Nevertheless, although we acknowledge that the response rate of our survey was low, the final number of respondents (more than 1 thousand) was considerable and, to our knowledge, higher than in any published study on the subject among established biomedical scientists.

The response rate of respondents in China was lower than for Europe. According to the first author’s personal experience and reported evidence [75], emails are not a common way for daily communication in China. We could have asked the 46 institutions to send the invitations to their staff members on our behalf, but this procedure would have entailed considerable administrative implications and, more importantly, respondents might have had doubts about the anonymity of the survey, which would have affected the reliability of responses. This is why we chose the more time and effort demanding option of sending individual emails to potential participants (except in the case of our own university), in the knowledge that this would be at the expense of a low response rate. Furthermore, our response rate is in the same order of magnitude as in the studies on research integrity or research environment among Chinese researchers by Liao et al. [30] and Han et al. [76].

In addition to the drawbacks above, we made efforts to improve the response rate. We kept the questionnaire brief and narrowed down our scope to plagiarism rather than asking about many different types of misconduct. Nevertheless, in light of the low response rate, we should be aware of the influence of non-response bias. Those who answered our questionnaire might have had a better understanding of plagiarism than those who did not. In addition, a large proportion of the respondents had international research experience, which might have influenced their perceptions of plagiarism. Therefore, our results might be biased towards “the best case”. In other words, if our survey revealed that well-educated and active biomedical researchers did not have an excellent understanding of plagiarism, we might presume that the understanding of other people may be even worse.

Although we developed the questionnaire on the basis of the Turnitin definition of plagiarism [27] and our previous work [28, 29], and carefully elaborated it, we acknowledge that it was not formally validated.

In the present work, we did not analyze the possible existence of differences in the perception of plagiarism within Europe, but such analyses are planned in the future.

### Practical implications

Our survey reveals that the understanding of plagiarism, especially knowledge of subtle forms of plagiarism, is still lacking among biomedical researchers based in European and Chinese top universities. We also observed a lower tendency to perceive several specific practices as plagiarism among China-based biomedical researchers compared to Europe-based researchers. Such lack of knowledge may increase the risk of plagiarism. In consequence, to improve the ability to recognize and avoid various forms of plagiarism, education and training about knowledge of plagiarism should be enhanced, and accompanied by giving concrete examples of plagiarism practices [23, 59, 61].

It is worth noting that the notion of plagiarism keeps developing and that boundaries of plagiarism have expanded a lot. More and more institutions and scholars start to discuss other forms of plagiarism, such as self-plagiarism [38, 77, 78]. In the future, as academic activity and intercultural communication increase, the understanding of plagiarism is very likely to be deepened.

In our opinion, avoiding plagiarism is more than just the avoidance of “copying and pasting”. It should be a reflection of good research practices. The best strategy for avoiding plagiarism, as well as promoting good research practices, should be increasing transparency, especially when there is doubt. In practice, always indicate the source of your words, images, ideas and probably other forms of “inspirations”.

## Conclusions

Through our online survey among biomedical researchers in leading universities in Europe and China, we found that these researchers had a generally good knowledge of the most obvious forms of plagiarism, but doubts with other practices still existed. To conclude, a clearer working definition is needed among biomedical researchers to deal with grey zones.

Despite largely similar responses among biomedical researchers based in Europe and China, a lower likelihood to perceive certain practices as plagiarism was observed among China-based researchers. This may indicate that the risk of facing plagiarism offenses could be greater in China than in Europe. We recognize that the relatively low number of respondents, as well as their possibly selected nature, affect the generalizability of our study, especially where no differences were observed between Chinese and European respondents. Nevertheless, even though our survey revealed some practices of plagiarism to be less likely recognized as such by (a minority of) Chinese respondents than by European respondents, it also suggested the existence of many similarities between both groups, thus leading us to the cautious conclusion that our findings should not be interpreted as “Chinese researchers perceive plagiarism differently from European researchers.” Our study represents a first endeavor to investigate empirically whether and how researchers in Europe and China differ in their perception of plagiarism.

## Supplementary information

**Additional file 1.** Survey on Perceptions of Plagiarism Definition. This file contains the questionnaire used in the online survey of this study.

**Additional file 2.** Universities included in the survey. This file contains the list of 46 universities that were selected in this study. The biomedical researchers in these universities were invited to participate in our online survey.

**Additional file 3.** Invitation to the online survey. This file contains the invitation letter sent to the selected respondents in this study.

**Additional file 4.** Consistency analysis of practices in pairs. This file contains the result of consistency analysis that was performed to investigate the consistency within each respondent between the responses to different questions in Section 2.

## Data Availability

The datasets used during the current study are available from the corresponding author on reasonable request.

## Abbreviation

FFP: fabrication, falsification and plagiarism
OR(s): odds ratio(s)
aOR(s): adjusted odds ratio(s)
CI(s): confidence interval(s)
SD: standard deviation
