# Supplementary material for: Perceptions of plagiarism by biomedical researchers: an online survey in Europe and China
Source: BMC Med Ethics. 2020 Jun 1;21:44. doi: 10.1186/s12910-020-00473-7 (PMC7268401; doi:10.1186/s12910-020-00473-7)
Supplement: Supplementary file 1 — Additional file 1. Survey on Perceptions of Plagiarism Definition. This file contains the questionnaire used in the online survey of this study. [file 12910_2020_473_MOESM1_ESM.docx]

**Additional file 1**

**Survey on Perceptions of Plagiarism Definition**

**Introduction**

We would like to know how the definition of plagiarism in biomedicine is perceived. Your collaboration will provide valuable data for us. The questionnaire distribution and data collection is performed by the online tool LimeSurvey, and so is completely anonymous, so please feel free to answer the questions. Filling out the survey counts as informed consent to participate in this study.

It takes about **5** minutes to go through all these questions. Thank you for your participation!

**Demographic information**

(Q1) Age (Q2) Gender 2

(Q3) Country of birth (Q4) Country where you work now 2

Q5. Your mother tongue is:

a. English b. Chinese c. Other

Q6. Current academic position:

a. Professor b. Associate professor c. Assistant professor d. PostDoc e. Other

Q7. If Q6=Other, What is your current academic position?

1

Q8. Do you have a PhD degree?

a. Yes 2

b. Not yet, I’m currently a PhD candidate

c. No

Q9. In which year did you obtain your PhD degree?

1

Q10. Have you done research for 6 months or more in another country than where you obtained your bachelor/master’s degree?

a. Yes b. No

Q11. If yes, in which country did you do that research?

1

**Section 1**

Please tick the most appropriate response(s).

Q12. **Plagiarism** is a greater threat to biomedical research than **data falsification**.

a. Strongly disagree b. Disagree c. Agree d. Strongly agree

Q13. **Plagiarism** is a greater threat to biomedical research than **granting co-authorship to someone whose contribution doesn’t justify it**.

a. Strongly disagree b. Disagree c. Agree d. Strongly agree

Q14. **Plagiarism** is a greater threat to biomedical research than **submitting a manuscript to more than one journals simultaneously**.

a. Strongly disagree b. Disagree c. Agree d. Strongly agree

Q15. Which factor(s) do you think decide whether a body of copied and unattributed text constitutes plagiarism or not?

a. The length of the copied text (whether a few sentences or paragraphs)

b. The part of the copied text (whether from the section of “background” or “results”)

c. The presence of an intention to copy without attribution

d. None of the above

Q16. Have you ever been unsure whether you are plagiarizing?

a. Yes b. No

**Section 2**

In your opinion, which of the following practices constitute(s) plagiarism?

Q17.

a. Copying text from someone else's publication without crediting the source.

b. Copying text from someone else's publication with crediting the source, but without quotation marks.

c. Copying text from someone else's publication with crediting the source and with quotation marks.

d. Copying an image from someone else's publication without crediting the source.

e. Using idea(s) from someone else's publication without crediting the source.

f. None of the above.

Q18.

a. Copying text from an online source without crediting the source.

b. Copying text from an online source that has no list of authors, and without crediting the source.

c. None of the above.

Q19.

a. Rephrasing another person’s work without crediting the source.

b. Rephrasing text from someone else's publication without significant modification of the original, but with crediting the source.

c. Summarizing another person’s work without crediting the source.

d. None of the above.

Q20.

a. Paying someone else to write a paper without granting authorship.

b. Having someone else to write a paper for free without granting authorship.

c. Putting together pieces from different publications, and presenting the result as one’s own work.

d. When writing a literature review, using the same framework of others’ review, without crediting the source.

e. With permission from the original author, using another’s text without crediting the source.

f. None of the above.

Q21.

a. Republishing others’ work in another language without crediting the source.

b. Republishing one’s own work in another language without crediting the source.

c. None of the above.

Q22.

a. Reusing one’s own previously rejected research proposal for another funding application without crediting the source.

b. Reusing a significant portion of one’s own previous publication for a new publication without crediting the source.

c. None of the above.

Q23. This is the last question of the survey.

a. One has submitted work as dissertation/thesis, and submits parts of it to a journal afterwards without crediting the source.

b. One has submitted work as dissertation/thesis, and submits a summary of it to a journal afterwards without crediting the source.

c. None of the above.

*Thank you again for spending your time to complete this survey!*
