# Supplementary material for: Perceptions of plagiarism by biomedical researchers: an online survey in Europe and China
Source: BMC Med Ethics. 2020 Jun 1;21:44. doi: 10.1186/s12910-020-00473-7 (PMC7268401; doi:10.1186/s12910-020-00473-7)
Supplement: Supplementary file 2 — Additional file 2. Universities included in the survey. This file contains the list of 46 universities that were selected in this study. The biomedical researchers in these universities were invited to participate in our online survey. [file 12910_2020_473_MOESM2_ESM.docx]

**Additional file 2**

**Universities included in the survey**

| Europe | China |
| --- | --- |
| Heidelberg University | Beihang University |
| Karolinska Institute^a^ | Beijing Institute of Technology |
| KU Leuven | Beijing Normal University |
| Leiden University | Central South University |
| University of Barcelona | Chongqing University |
| University of Cambridge | Dalian University of Technology |
| University of Edinburgh | East China Normal University |
| University of Geneva | Fudan University |
| University of Helsinki | Harbin Institute of Technology |
| University of Milan | Huazhong University of Science and Technology |
| University of Munich | Jilin University |
| University of Oxford | Lanzhou University |
| University of Strasbourg | Minzu University of China |
|  | Nanjing University |
|  | Nankai University |
|  | Northwestern Polytechnical University |
|  | Ocean University of China |
|  | Peking University |
|  | Shandong University |
|  | Shanghai Jiao Tong University |
|  | Sichuan University |
|  | South China University of Technology |
|  | Southeast University |
|  | Sun Yat-sen University |
|  | Tianjin University |
|  | Tongji University |
|  | Tsinghua University |
|  | University of Electronic Science and Technology of China |
|  | University of Science and Technology of China |
|  | Wuhan University |
|  | Xi'an Jiaotong University |
|  | Xiamen University |
|  | Zhejiang University |

^a^ Former member of LERU.
