# Supplementary material for: Perceptions of plagiarism by biomedical researchers: an online survey in Europe and China
Source: BMC Med Ethics. 2020 Jun 1;21:44. doi: 10.1186/s12910-020-00473-7 (PMC7268401; doi:10.1186/s12910-020-00473-7)
Supplement: Supplementary file 3 — Additional file 3. Invitation to the online survey. This file contains the invitation letter sent to the selected respondents in this study. [file 12910_2020_473_MOESM3_ESM.docx]

**Additional file 3**

**Invitation to the online survey**

The English invitation was sent to Europe-based researchers. An invitation in the language of both English and Chinese was sent to China-based researchers.

*English version*

Subject: Invitation to the survey of plagiarism definition

Dear researcher:

Several weeks ago, based on the public email addresses on the website of your esteemed university, we sent you an invitation to the survey of plagiarism definition. The survey aims to investigate perceptions of plagiarism definition among biomedical researchers (including researchers active in domains of medicine, pharmaceutical science and life sciences, etc.) in top universities in Europe and China.

Sorry for interrupting again, and this will be the last reminder. We are still interested in your answer. Please ignore this email if you have completed the survey. If not, we sincerely invite you to spend 5 minutes to participate in our survey, to provide valuable answers for us! You can easily start it by clicking the following link. Anonymity will be guaranteed. The questionnaire distribution and collection is completely performed by the online survey tool LimeSurvey. All the information is to be used for research only.

https://websurvey.kuleuven.be/index.php/969964/lang-en

The deadline is April 15^th^ (for the first-round invitation) /May 30^th^ (for the second-round invitation), 2018. The access of the questionnaire will be closed after that date. You only need to fill in the survey once. The survey is available in both English and Chinese.

In case of any questions of this survey, please contact: nannan.yi@kuleuven.be.

Thank you very much for your participation!

We wish you all the best with your research!

Yours sincerely,

Prof. Kris Dierickx, Prof. Benoit Nemery, Dra Nannan Yi (PhD researcher)

Centre for Biomedical Ethics and Law

Faculty of Medicine

KU Leuven

(For more information on our projects and publication on this topic, see https://www.kuleuven.be/wieiswie/en/person/00014957)

*Chinese version*

主题：诚邀您参与有关剽窃概念的调查

尊敬的老师：

您好！

几周前，我们根据贵校官网上公布的邮箱，冒昧地给您发出了一个有关剽窃概念的调查，旨在了解中国和欧洲顶尖高校的生物医学科研工作者（包括医学、药学、生命科学等领域的科研人员）对剽窃概念的理解。

抱歉再次打扰您，我们仍然对您的回答很感兴趣，并且这是我们的最后一次提醒。如果您已经完成问卷，请忽略此邮件。如果您尚未参与，我们再次诚挚地邀请您花5分钟的时间为我们的研究提供宝贵的信息！您只需要点击以下链接即可开始。所有回答都是严格匿名的，我们无从确认您的身份。问卷发放和回收均由在线调查工具LimeSurvey完成，所有信息也仅用于科研，绝不会用于他处。

https://websurvey.kuleuven.be/index.php/969964/lang-zh-Hans

调查截止日期为2018年4月15日（第一轮邀请）/5月30日（第二轮邀请），随后本问卷答题页面将会关闭。问卷有中/英文版本，您仅需作答一次。

若您有关于本次调查的任何困惑或疑问，可直接联系邮箱：nannan.yi@kuleuven.be。

感谢您的参与！

祝愿您科研工作一切顺利！

Kris Dierickx教授、Benoit Nemery教授、博士研究生乙楠楠

比利时鲁汶大学医学伦理与法律中心

(关于我们课题和成果的更多信息，请见https://www.kuleuven.be/wieiswie/en/person/00014957)
