# Supplementary material for: Perceptions of plagiarism by biomedical researchers: an online survey in Europe and China
Source: BMC Med Ethics. 2020 Jun 1;21:44. doi: 10.1186/s12910-020-00473-7 (PMC7268401; doi:10.1186/s12910-020-00473-7)
Supplement: Supplementary file 4 — Additional file 4. Consistency analysis of practices in pairs. This file contains the result of consistency analysis that was performed to investigate the consistency within each respondent between the responses to different questions in Section 2. [file 12910_2020_473_MOESM4_ESM.docx]

**Additional file 4**

**Table S4.1 Consistency analysis of practices in pairs**

|  | **Practice 1** | **Practice 2** | **Kappa coefficient (95%CI)** |
| --- | --- | --- | --- |
| **Question 17** | Copying text from someone else's publication without crediting the source. | Copying text from someone else's publication with crediting the source, but without quotation marks. | 0.025  (0.007;0.043) |
|  | Copying text from someone else's publication without crediting the source. | Copying text from someone else's publication with crediting the source and with quotation marks. | -0.001  (-0.007;0.004) |
|  | Copying text from someone else's publication without crediting the source. | Copying an image from someone else's publication without crediting the source. | 0.160  (0.031;0.289) |
|  | Copying text from someone else's publication without crediting the source. | Using idea(s) from someone else's publication without crediting the source. | 0.024  (-0.005;0.052) |
|  | Copying text from someone else's publication with crediting the source, but without quotation marks. | Copying text from someone else's publication with crediting the source and with quotation marks. | 0.085  (0.055;0.116) |
|  | Copying text from someone else's publication with crediting the source, but without quotation marks. | Copying an image from someone else's publication without crediting the source. | 0.026  (0.003;0.049) |
|  | Copying text from someone else's publication with crediting the source, but without quotation marks. | Using idea(s) from someone else's publication without crediting the source. | 0.010  (-0.047;0.067) |
|  | Copying text from someone else's publication with crediting the source and with quotation marks. | Copying an image from someone else's publication without crediting the source. | -0.008  (-0.017;0.002) |
|  | Copying text from someone else's publication with crediting the source and with quotation marks. | Using idea(s) from someone else's publication without crediting the source. | 0.008  (-0.012;0.029) |
|  | Copying an image from someone else's publication without crediting the source. | Using idea(s) from someone else's publication without crediting the source. | 0.020  (-0.015;0.055) |
| **Question 18** | Copying text from an online source without crediting the source. | Copying text from an online source that has no list of authors, and without crediting the source. | 0.170  (0.106;0.233) |
| **Question 19** | Rephrasing another person’s work without crediting the source. | Rephrasing text from someone else's publication without significant modification of the original, but with crediting the source. | 0.008  (-0.014;0.030) |
|  | Rephrasing another person’s work without crediting the source. | Summarizing another person’s work without crediting the source. | 0.399  (0.329;0.469) |
|  | Rephrasing text from someone else's publication without significant modification of the original, but with crediting the source. | Summarizing another person’s work without crediting the source. | 0.020  (-0.005;0.046) |
| **Question 20** | Paying someone else to write a paper without granting authorship. | Having someone else to write a paper for free without granting authorship. | **0.712**  **(0.670;0.754)** |
|  | Paying someone else to write a paper without granting authorship. | Putting together pieces from different publications, and presenting the result as one’s own work. | 0.043  (0.024;0.063) |
|  | Paying someone else to write a paper without granting authorship. | When writing a literature review, using the same framework of others’ review, without crediting the source. | 0.188  (0.131;0.244) |
|  | Paying someone else to write a paper without granting authorship. | With permission from the original author, using another’s text without crediting the source. | 0.140  (0.091;0.189) |
|  | Having someone else to write a paper for free without granting authorship. | Putting together pieces from different publications, and presenting the result as one’s own work. | 0.026  (-0.002;0.054) |
|  | Having someone else to write a paper for free without granting authorship. | When writing a literature review, using the same framework of others’ review, without crediting the source. | 0.140  (0.080;0.201) |
|  | Having someone else to write a paper for free without granting authorship. | With permission from the original author, using another’s text without crediting the source. | 0.141  (0.084;0.197) |
|  | Putting together pieces from different publications, and presenting the result as one’s own work. | When writing a literature review, using the same framework of others’ review, without crediting the source. | 0.040  (0.010;0.071) |
|  | Putting together pieces from different publications, and presenting the result as one’s own work. | With permission from the original author, using another’s text without crediting the source. | 0.073  (0.030;0.117) |
|  | When writing a literature review, using the same framework of others’ review, without crediting the source. | With permission from the original author, using another’s text without crediting the source. | 0.172  (0.115;0.230) |
| **Question 21** | Republishing others’ work in another language without crediting the source. | Republishing one’s own work in another language without crediting the source. | 0.017  (-0.005;0.040) |
| **Question 22** | Reusing one’s own previously rejected research proposal for another funding application without crediting the source. | Reusing a significant portion of one’s own previous publication for a new publication without crediting the source. | 0.035  (0.017;0.053) |
| **Question 23** | One has submitted work as dissertation/thesis, and submits parts of it to a journal afterwards without crediting the source. | One has submitted work as dissertation/thesis, and submits a summary of it to a journal afterwards without crediting the source. | **0.782 (0.740;0.825)** |
